# The Role of Genetic Selection on Agonistic Behavior and Welfare of Gestating Sows Housed in Large Semi-Static Groups

**DOI:** 10.3390/ani10122299

**Published:** 2020-12-04

**Authors:** Sophie Brajon, Jamie Ahloy-Dallaire, Nicolas Devillers, Frédéric Guay

**Affiliations:** 1 Department of Animal Science, Faculty of Agriculture and Food Sciences, Laval University, 2425 Rue de l’Agriculture Bureau 1122, Québec, QC G1V 0A6, Canada; Jamie.Ahloy-Dallaire.1@ulaval.ca (J.A.-D.); Frederic.Guay@fsaa.ulaval.ca (F.G.); 2 Sherbrooke Research and Development Centre, Agriculture and Agri-Food Canada, 2000 College Street, Sherbrooke, QC J1M 0C8, Canada; Nicolas.Devillers@canada.ca

**Keywords:** swine, *Sus scrofa*, genetic line, group-housing, social relationships, aggression, body lesion, productivity, pig industry

## Abstract

**Simple Summary:**

Group-housing of gestating sows is becoming increasingly common worldwide as it offers the sows the opportunity to exercise, display exploratory behaviors, and develop social relationships. Despite its advantages, group-housing as it stands in modern industries also presents several welfare issues such as overt aggression between pen-mates and resulting stress and injuries. To date, breeding companies often largely focused their efforts on genetic selection based on individual production characteristics (e.g., litter size, piglets’ growth, and meat quality) and traditionally ignored the social behaviors and the ability to establish a dominance hierarchy without exacerbated aggression. Hence, the extent to which agonistic behavior differs according to new genetic lines is unknown. The objective of this study was to compare and investigate the influence of two genetic lines on the welfare and performance of sows housed in large semi-static groups (up to 91 animals). While the first genetic line was more aggressive toward pen-mates during gestation, the second had piglets with a lower robustness and survivability. This study raises the difficulty of finding an optimal genetic line, including both positive welfare and productivity outcomes, and points to the urgent need of considering social aspects when developing genetic lines for group-housing.

**Abstract:**

Confinement of gestating sows is becoming banished in favor of group-housing in countries worldwide, forcing breeding companies to develop genetic lines adapted for social living. This study aimed at assessing the influence of two genetic lines selected for high performance (HP1, HP2, derived from Landrace × Yorkshire) on welfare and reproductive performance of sows housed in large semi-static groups (20 groups of 46–91 animals) across several parities. To address this, agonistic behaviors were recorded on d0, d2, d27, and d29 post-mixing while body lesions were scored on d1, d26, and d84. Sows’ individual and reproductive performances were also recorded. HP2 sows were more aggressive than HP1 sows since they fought (*p* = 0.028) and bullied (*p* = 0.0009) pen-mates more frequently on d0–d2. HP2 sows had more total body lesions throughout gestation than HP1 sows at higher parities (*p* < 0.0001). Regarding reproductive performance, HP2 sows lost less piglets (*p* < 0.0001) and tended to wean more piglets (*p* = 0.067) than HP1 sows. In conclusion, while HP2 sows were the most aggressive, HP1 sows had piglets with lower survivability, which raises ethical issues in both cases and points to the need of considering social aspects when developing genetic lines for group-housing.

## 1. Introduction

With increasing public awareness regarding pig welfare, gestating sow housing systems are currently changing around the world to group-housing. While the legislation on sows’ group-housing was adopted in 2001 in Europe, the new Canadian regulation was enacted in 2014. The Canadian Code of Practice for the care and handling of pigs requires that all newly built facilities or those undergoing renovation must house sows in groups during gestation, or provide means that allow greater freedom of movement, and the code currently states that this regulation must be effective to all pig farms by 2024 [1]. Group-housing undoubtedly has advantages in favor of animal welfare; pregnant sows have the opportunity to exercise, display exploratory behavior, but also develop social relationships with conspecifics [2]. As social animals, pigs value familiar conspecifics and they are motivated to work in order to have access to a social partner [3]. Despite its advantages [2], group housing as it stands in modern industries (i.e., management practice of frequently mixing animals and reforming groups) presents several potential welfare issues (social instability, agonistic behavior, and injuries). For instance, fights that occur to establish social hierarchy can be very intense and result in severe body lesions and lameness [4] where the most vulnerable animals can become unable to walk. This can imply reduced productivity (weaned piglets per sow) and higher production costs [4,5]. Extensive research has been done in recent decades to identify best housing strategies to improve welfare and production performance, with a reduced cost. However, aggressiveness resulting from mixing unfamiliar sows is still a serious issue in pig farming, causing stress, injuries, lameness, and reproduction failure [6,7].

Improving social and housing conditions or modifying herd management practices are among the strategies explored to decrease aggressiveness [8,9,10]. Another lever for action is through genetic selection against aberrant and agonistic behaviors [9], but also for good social skills. However, to date, pig breeding companies have often largely focused their efforts on genetic selection based on individual production characteristics [11] rather than on welfare and adaptability of animals to farming conditions. Several swine studies indicated that aggressiveness and social interactions have a moderate level of heritability in terms of risk for body lesions and fighting behavior expression [12,13,14]. These traits related to well-being may thus be selected inadvertently, as a consequence of selection for highly productive animals [15]. For instance, Breuer et al. [16] reported that Durocs were more prone to tail-biting of pen-mates than Large White and Landrace pigs whereas Landrace belly-nosed pen-mates more often, showing evidence of a genetic basis for the expression of harmful social behaviors. In addition, Chu et al. [15] explored the relationship between breed and aggressiveness in two-month-old female pigs and they found that Chinese indigenous Mi pigs were less aggressive than cross bred Landrace-Large white (LLW) pigs.

Paradoxically, ignoring the effects of social interactions between pen-mates in breeding programs (e.g., by evaluating breeding potential in individually-housed sows) might cause biased estimates and suboptimal responses to selection, which may eventually have deleterious effects on productivity [17]. Indeed, the challenges animals experience in their life change their physiology and can induce reallocation of energy on body maintenance or anxiety-related behaviors at the expense of reproductive performance [6,18,19]. This was observed in laying hens where the selection for high performance increased competition and aggressiveness between individuals, resulting in reduced productivity of pen-mates [20]. Social stress in group-housed sows, one of the main challenges in their life, has been found to increase the risk of abortion [7], alter fetal development [21], impair maternal abilities [22], and affect piglet growth [23] and behavior [24] depending on the gestational period.

The evaluation of genetic lines in social housing conditions as found in pig industries is thus essential to apprehend the indirect effect of behavior on welfare and productivity. Most of the previous work was performed under highly controlled conditions and often with small herd size, while livestock farming faces structural changes and increased herd sizes. Hence, the present study aimed at evaluating the influence of genetic line on agonistic behavior, welfare, and individual performance of sows housed in semi-static large groups in a commercial setting. Agonistic behavior and body lesions, a validated proxy measure of aggression [13], were recorded around mixing and later during gestation. It was hypothesized that two separate processes of selection for reproductive and other performance characteristics, within a same breed but carried out by two different breeding companies, induced behavioral differences between genetic lines and modulated welfare differently.

## 2. Materials and Methods 

### 2.1. Ethical Note

All procedures on animals were approved by the Laval University (Quebec City, QC, Canada, approval number: 2018102-1) institutional animal care committee, and the farm where the study was performed was in compliance with the guidelines set out in the Canadian Code of practice for the care and handling of pigs [1]. Sow and piglet health was monitored daily on the afternoons throughout the experiment and animals were removed from the group if showing moderate to severe lameness or suffering from significant injury or illness.

### 2.2. Animal Treatments and Management

The treatment consisted of two pre-existing genetic lines selected for high performance, HP1 and HP2 lines, which were both F1 derived from Landrace × Yorkshire sows. The two genetic lines were developed by two independent Canadian suppliers, but the breeding programs had some similarities (Table 1), even if the detailed set of criteria differed between the suppliers. Although social aspects were not part of a formal selection criterion in the breeding scheme, they were considered in HP2 but not in HP1. More particularly, the common practice in HP2 breeding company was to remove sows difficult to handle or causing important distress to pen mates, an issue that concerned a minority of animals. The experiment took place in a gestation unit of a commercial piggery located in Chaudière-Appalaches (Quebec, QC, Canada) between February 2019 and February 2020. 

The piggery was designed to raise an average of 900 heads divided in five cohorts (two groups per cohort). Cohorts of sows were synchronized so that one cohort was inseminated every 28 days.

This study used 645 gestating sows (319 from HP1; 326 from HP2) of mixed parities (range 1–5) and was conducted over 10 replicates (Table S1). Each replicate involved one group of each genetic line and the experimental unit was the group (N_HP1_ = 10; N_HP2_ = 10). Groups studied were part of the third to the fifth rotation. The rotation number corresponded to the insemination number (e.g., rotation 3 = third insemination of the group) but sows within the group could have a lower parity (up to two gestations late) if they had previous reproductive failures and were moved to the next group. Group sizes decreased across time and ranged from 91 to 44 animals since no further animals were bought from the groups acquisition. The mean (±SE) group size was 74.2 ± 5.0, 55.8 ± 3.0 and 52.0 ± 3.3 in rotations 3, 4, and 5, respectively.

After acquisition from the suppliers, sows were raised in groups of the same genetic line after weaning in the same fattening unit until an average of 205 days old (135–140 kg) and then moved to the gestating unit where they were inseminated in individual insemination stalls (1.83 m × 0.61 m) prior to mixing. While in insemination stalls, sows were fed using a manual feed dropper above the feed trough once daily in the morning. All diets were formulated to meet the sows’ nutritional requirement according to the National Research Council [25] and the flushing diet contained 15.1% min. crude protein, 4.5% min. crude fat, 3.0% max. crude fiber as fed basis (i.e., equivalent to 17.4% min. crude protein, 5.2% min. crude fat and 3.5% min. crude fiber as Dry Matter (DM, 87%) basis).

At 5–7 days post-insemination, sows were moved from their insemination stalls and mixed with other sows of the group from the same genetic line in one of the six large gestation pens (day of mixing: d0). Each pen was 9.45 m long and 21.34 m wide (total of 201.66 m^2^), which provided at least 2.21 m^2^ floor space allowance per sow. Pens had partly slatted concrete flooring with four plastic walls in a central area and five solid concrete resting areas (6.89 m^2^ each) separated by concrete walls in the back area. While in gestation pens, sows were fed using an Electronic Sow Feeder (ESF, Compident ESF, Schauer Agrotronic GmbH, Prambachkirchen, Austria) located at the front of the pens. The ESF opened at 2200 and closed once all the sows were fed or one hour after the last visit if one or two sows did not feed. When more than two sows did not feed, the ESF closed at 1800 if there were no visits since at least one hour. Sow feeding was monitored daily by farm personnel using the ESF monitoring software records (FarmManager software, Schauer Agrotronic GmbH, Prambachkirchen, Austria) and instances of off-feed events were noted. Sows that did not eat the previous day were examined and encouraged to enter the ESF using a panel, if needed. Throughout gestation, sows were fed with a diet containing 12.5% min. crude protein, 3.5% min. crude fat, 6.6% max. crude fiber as fed basis (i.e., equivalent to 14.2% min. crude protein, and 4.0% min. crude fat, and 7.5% min. crude fiber as DM (88%) basis) [25]. Sows were provided water ad libitum from one of the three water bowls and four nipple drinkers in the pens.

Pregnancy diagnosis was performed using two methods during the first weeks of gestation. Sows which showed behavioral signs of estrus during the first two weeks after mixing were directly removed from the gestation pen and transferred to the insemination stalls to be re-inseminated and added to the following group of the same genetic line (cohort +1). In addition, pregnancy tests using ultrasound scanning were performed at d23 post-mixing. Sows that tested negative in the pregnancy test were then removed from the group at d28 post-mixing (i.e., group reduction day) and housed in individual insemination stalls until the following insemination phase one month later (cohort +2). The gestating sows remained in the gestation pen for 102 days after mixing.

One week prior to farrowing, sows were moved to individual farrowing crates (1.52 m × 2.13 m) where they remained until weaning. Sows were fed with a diet including 19.4% min. crude protein, 4.7% min. crude fat, 3.3% max. crude fiber as fed basis (i.e., equivalent to 22.0% min. crude protein, 5.3% min. crude fat, and 3.7% min. crude fiber as DM (88%) basis) [25] using an individual automatic feeder (Gestal Quattro, Jyga Technologies Inc.©, St-Lambert-de-Lauzon, QC, Canada) in the farrowing crates. Lights were on from 0700 to 1900, temperature was maintained between 19.2 and 22.2 °C and rooms were mechanically ventilated throughout the piggery.

### 2.3. Data Collection

#### 2.3.1. Agonistic Behavior

Two digital HD video camera recorders (Sony CX455 Handycam^®^, Tokyo, Japan) were installed at two opposite corners of the pens and recorded at 15 frames per second from 0900 to 1600 h on the day of mixing (d0), and from 0700 to 1130 h on day 2 post-mixing (d2), the day before group reduction (d27) and the day after group reduction (d29). Although the cameras covered most of the pen floor area, some floor area in the corners of the pen, which corresponded to the back of the resting areas, was blind. However, the central open area and the area around the ESF, where most of the aggressive encounters occurred, were within the field of view of the cameras. The use of non-competitive ESF systems (i.e., offering full protection to the sow at feeding, [26]), prevented exacerbated aggression at feeding. Animals were fed throughout the night and part of the day, allowing for the establishment of a feeding order, which has been previously demonstrated [27]. Preliminary observations indicated that most of the aggression was delivered when lights turned on in the morning, and therefore it was decided to analyze agonistic behavior at this sampling time.

All the sows were individually identified by colour mark coding on the back, in order to determine the identities of the perpetrator and recipient of agonistic behaviors. When the colour mark was unidentifiable, the sow was referred to as unidentified, but the identifiable opponent was noted correctly. Sow agonistic behavior was coded continuously from the video recordings for 4 h post-mixing, and for 2 h from 0700 at days 2, 27, and 29 post-mixing using the video analysis software The Observer XT 14.2 (Noldus Information Technology, Wageningen, The Netherlands). Behaviors recorded as non-reciprocal agonistic acts were threats (the perpetrator suddenly stretches the neck toward a recipient and provokes the recipient to avoid or escape without physical contact), bites and knocks (the perpetrator bites or gives a head knock to the recipient) and bullying (the perpetrator gives a series of three or more bites and/or knocks to the recipient). Fights and their durations were also recorded, defined as a reciprocal interaction of at least three bites or knocks where each opponent bites or gives a head knock at least once and for a total duration of at least 3 s [28,29]. The identities of the initiator (sow which gives the first agonistic act), the receiver (sow which receives the first agonistic act), the winner (sow which gives the last agonistic act) and the loser (sow which avoids or escapes after the last agonistic act) were recorded. When there was no clear winner or loser, the fight outcome was considered as undetermined.

All video analyses were performed by a single trained experimenter and the percentage of intra-observer agreement was calculated using 20 min video sequences per group for a total of 400 min. There was agreement (agreement = 1) when data from both observations were identical and disagreement (agreement = 0) when a data point was missing, in excess or different from the data of the second observation. A tolerance of 1-s difference was chosen for the intra-observer agreement between durations. The intra-observer agreement was 91.6%, 89.5%, and 94.0% for sow identity, behavior and fight duration, respectively. Groups were identified by a unique code visible on the cameras allowing the observer to be blind to treatments.

#### 2.3.2. Body Lesions

Body lesions of all the sows were recorded on the day before mixing (d-1), the day after mixing (d1), two days before group reduction (d26), and after three months of gestation (d84). At d-1 and d26, sows were individually identified using a colour mark coding on the back immediately after lesion scoring. The first body lesion scoring (d-1) served as baseline and was subtracted from the body lesion scoring at d1. While the first lesion scoring at d-1 was performed in the individual insemination stall, the next lesion scorings were performed in the gestation pens.

The method used to score body lesions was adapted from Turner et al. [30] and Calderón Díaz et al. [31]. Three body regions were examined on the left and right sides for a total of six scores per scoring day: front (head, neck, shoulders, and front legs), middle (back and flanks), and rear (rump and hind legs). Body lesions for each region and side were scored as follow: 0 = None or 1 superficial lesion; 1 = 2 to 5 superficial lesions; 2 = more than 5 superficial lesions and/or 1 slightly deep red lesions of 2 to 5 cm length; 3 = 2 to 5 slightly deep red lesions of 2 to 5 cm length and/or 1 very deep red lesion of more than 5 cm length; 4 = more than 5 slightly deep red lesions of 2 to 5 cm length and/or 2 to 5 very deep red lesions of more than 5 cm length; and 5 = more than 5 very deep red lesions of more than 5 cm length.

The body lesion scoring at d84 of the last replicate that had to be recorded in March 2020 was missing due to prohibited access to the farm, as a safety measure, during the worldwide COVID-19 pandemic. The body lesion scores were recorded by a single trained experimenter and the intra-observer agreement was evaluated using body lesion scoring from 305 sows randomly selected at different points of the experiment which were scored twice on the same scoring day (intra-observer agreement: 93.2%).

#### 2.3.3. Individual and Reproductive Performance

Backfat thickness and body weight of randomly selected sows were recorded as indicators of individual performance. Selected sows were subjected to backfat thickness measurements before insemination (N_obs_ = 204) and/or before farrowing (N_obs_ = 449). In addition, these same or different selected sows were weighed before farrowing (N_obs_ = 550) and/or after weaning (N_obs_ = 532).

Success to farrowing (i.e., sows that successfully give birth to at least one alive piglet, N_obs_ = 1242) was recorded for all sows and regarded as an indicator of reproductive performance. Additional data were also recorded for the sows randomly selected for individual performance measurements. Additional collected data included the litter size (number of total born piglets, N_obs_ = 592), number of piglets born alive (N_obs_ = 592), stillborn (N_obs_ = 592), mummified (N_obs_ = 592), total dead (N_obs_ = 563), and weaned (N_obs_ = 578) as well as the litter weight of alive piglets at birth (N_obs_ = 592).

### 2.4. Statistical Analyses

Analyses were carried out using SAS software (version 9.2; SAS Institute Inc., Cary, NC, USA). Data were analyzed using the sow within the group as the repeated factor, except performance data where the repeated factor was the sow. For continuous data, normality of model residuals was tested using the Shapiro–Wilk test of normality.

The effect of genetic line on aggression behavior was analyzed using multivariate linear models. Observation duration differed according to observation day (i.e., four hours at mixing, two hours at d2, d27, and d29) so behavior frequency and total duration were divided by the number of observed hours to give behavior frequency and duration per hour. Group size decreased across time, since no further animals were bought from the groups acquisition, and was confounded with rotation and parity, which increased across time. Hence, the rotation variable included in the analyses was regarded as an integrative variable taking into account parity and group size. Although group size varied across time, it did not differ between genetic lines (Mann–Whitney U-test: $\bar{X}$_HP1_ *± SE* = 62.1 ± 4.6, $\bar{X}$_HP2_ *± SE* = 62.0 ± 4.8, *U* = 104.5, *p* = 0.493). Independent variables considered as fixed effects were genetic line (HP1, HP2), rotation (3, 4, 5) and day (d0, d2, d27, d29) while replicate (1 to 10) was considered as random effect. The variables of rotation and observation day were removed from the model if non-significant. Otherwise, interactions were tested and kept when significant. Because data distributions were skewed, the number of agonistic acts were analyzed using the GLIMMIX procedure with Poisson distribution and log link function, while fight durations were analyzed using the GLIMMIX procedure with lognormal distribution and identity link function. The denominator degrees of freedom of these latter analyses were computed using the Satterthwaite approximation. Multiple comparisons were performed using Tukey’s adjustment of the Student’s *t* tests. The estimates were then back-transformed to the original scale and model results were expressed as predictive means (CI).

Lesion scores from the left and right sides of the body were pooled and averaged to keep only one score for the front, middle and rear part of the body. In addition, the six body lesion scores were pooled and averaged to create the variable total body lesions. Thereafter, the body lesion scores were re-categorized in four balanced categories for each body region to be properly analyzed (very low, low, high, and very high). Those ordinal categorical variables were then modelled using multinomial models to fit cumulative logit proportional odds model to the data and odds ratios were calculated. Independent variables considered as fixed effects were genetic line, rotation, and day (d1 adjusted, i.e., lesion scores at d1–lesion scores at d-1), d26, and d84) while replicate (1 to 10) was considered as random effect. Multiple comparisons were performed using Tukey’s adjustment of the Student’s *t* tests. Thereafter, Spearman’s rank correlations were performed to investigate whether body lesion scores were associated with agonistic behaviors. To do this, Spearman’s rho were calculated by rotation and genetic and weighted sum of Spearman’s rhos were computed for testing the association between variables by taking into account rotation and genetic as blocking variables [32].

Individual and reproductive performances were analyzed by multivariate linear models with genetic line and rotation considered as fixed effects and replicate considered as random effect. Residuals from most of the performance analyses had a normal distribution except the number of piglets stillborn which was modelled with a Poisson distribution and the total number of dead piglets which was modelled with a lognormal distribution. The variables success to farrowing and presence of mummified piglets were binary and were modelled using mixed logistic regression. Model results were expressed as predictive means ± standard errors.

## 3. Results

### 3.1. Agonistic Behavior

**Figure 1:**
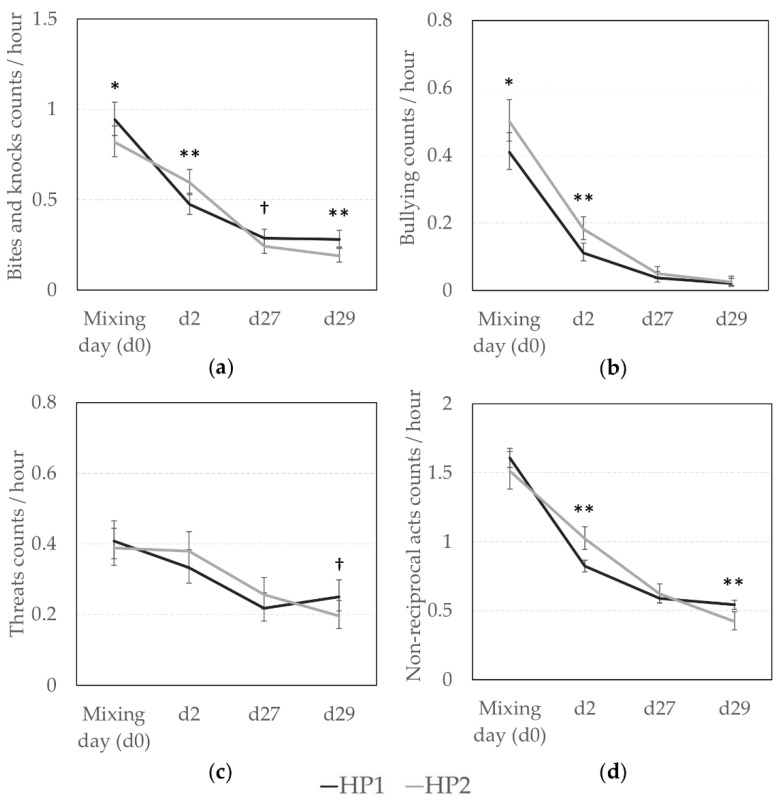
Non-reciprocal agonistic behaviors according to the genetic line (HP1, HP2). Back-transformed predicted mean (CI) number of (**a**) bites and knocks, (**b**) bullying, (**c**) threats, (**d**) total non-reciprocal acts (bites, knocks, bullying, and threats) given per hour at d0 (mixing), d2, d27, and d29. *, **; Means differ significantly (* *p* < 0.050, ** *p* < 0.010); † Means tend to differ (*p* < 0.10) between genetic lines for a same observation day.

**Figure 2:**
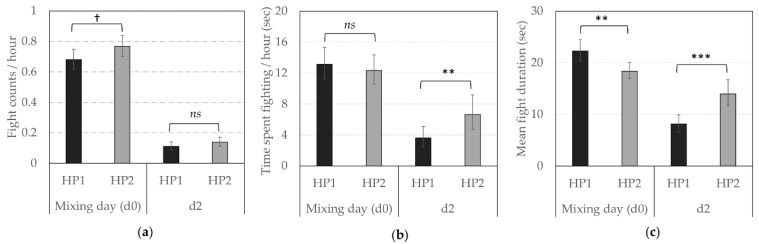
Reciprocal agonistic behaviors according to the genetic line (HP1, HP2). Back-transformed predicted mean (CI) (**a**) number of fights per hour, (**b**) time spent fighting per hour (in sec) and (**c**) average fight duration (in sec) on d0 (mixing) and d2. **, ***; Means differ significantly (** *p* < 0.010, *** *p* < 0.001); † Means tend to differ (*p* < 0.10) between genetic lines for a same observation day; *ns*—not significant.

Results for non-reciprocal and reciprocal agonistic behaviors are presented in Figure 1 and Figure 2, respectively. Overall, sows from the HP2 line had a more aggressive behavioral pattern around mixing, but the effect of genetic line differed according to the day of observation. HP1 sows gave more bites and knocks at mixing (Figure 1a, *F*_1,3393_ = 3.91, *p* = 0.048), but HP2 sows were more involved in those agonistic behaviors on d2 (*F*_1,3393_ = 7.13, *p* = 0.008). In total, HP2 gave more non-reciprocal acts (i.e., bites, knocks, threats, and bullying) at d2 than HP1 sows (Figure 1d, *F*_1,3399_ = 9.41, *p* = 0.002). In addition, HP2 sows initiated more bullying independently of the observation day (Back-transformed predictive means (CI): 0.10 (0.09; 0.12) vs. 0.08 (0.07; 0.09) per hour, *F*_1,3402_ = 11.04, *p* = 0.0009, Figure 1b) even if bullying frequency decreased across time (Back-transformed predictive means (CI): 0.45 (0.41; 0.49), 0.14 (0.12; 0.17), 0.04 (0.03; 0.06), and 0.02 (0.01; 0.03) per hour on d0, d2, d27, d29, respectively, *F*_1,3402_ = 194.48, *p* < 0.0001).

Regarding the reciprocal agonistics acts, HP2 sows were involved in more fights around mixing (i.e., d0 and d2), independently of the day (Back-transformed predictive means fight frequency/hour (CI) between d0 and d2: 0.32 (0.29; 0.36) vs. 0.28 (0.25; 0.31) per hour, *F*_1,1240_ = 4.82, *p* = 0.028, Figure 2a), but the mean number of fights per hour drastically decreased between mixing and d2 (Back-transformed predictive means (CI): 0.72 (0.68; 0.77) vs. 0.12 (0.11; 0.15) per hour, *F*_1,1240_ = 408.80, *p* < 0.0001). The total time spent fighting at mixing did not significantly differ between sows from both genetic lines but was higher for HP2 on d2 (Figure 2b, *F*_1,870.4_ = 9.80, *p* = 0.002). The mean duration of fights was higher for HP1 at mixing and higher for HP2 on d2 (Figure 2c, *F*_1,1123_ = 8.26, *p* = 0.004 and *F*_1,979.1_ = 16.41, *p* < 0.0001 on d0 and d2, respectively).

On days 27 and 29, agonistic interactions were rather rare for both genetic lines, but some effects were still visible. HP1 sows tended to give more bites and knocks than HP2 sows on day 27 and they gave significantly more bites and knocks on day 29 (*F*_1,3393_ = 2.72, *p* = 0.099, and *F*_1,3393_ = 10.78, *p* = 0.001, respectively). They also tended to give more threats on day 29 (Figure 1c, *F*_1,3393_ = 3.67, *p* = 0.056). In total, they were more involved in non-reciprocal agonistic acts at d29 (*F*_1,3399_ = 7.34, *p* = 0.007). Incidences of fighting during this time period were minimal, and thus statistical analyses were not conducted.

Agonistic behavior also varied between rotations and this variable was included in the analyses when significant. Overall, sows from rotation 5 tended to give more bites and knocks (*F*_2,3393_ = 2.37, *p* = 0.093) and threatened pen-mates more (*F*_2,3393_ = 54.97, *p* < 0.0001) at mixing, whereas sows from rotation 4 tended to give the most bites and knocks to pen-mates (*F*_2,3393_ = 2.88, *p* = 0.056) and they gave more threats (*F*_2,3393_ = 7.62, *p* = 0.0005) on d2. One month later, sows from rotation 3 were the most aggressive since they gave more bites/knocks and threats than sows from the other rotations at d27 (*F*_2,3393_ = 44.28, *p* < 0.0001, and *F*_2,3393_ = 14.04, *p* < 0.0001, respectively) and d29 (*F*_2,3393_ = 19.44, *p* < 0.0001, and *F*_2,3393_ = 6.93, *p* < 0.001, respectively).

### 3.2. Body Lesions

The effect of genetic line on body lesion scores was significant. However, there was a significant triple interaction effect between genetic line × rotation × scoring day on front, rear, and total body lesion scores (*F*_2,2089_ = 2.83, 2.21, 2.04, and *p* = 0.010, 0.040, 0.057, respectively) and a genetic line × rotation interaction effect on middle body lesion scores (*F*_2,2089_ = 28.47, *p* < 0.0001). Thus, analyses were performed a second time by rotation and results from genetic line, scoring day and their interaction effect are presented in Table 2, Table 3, Table 4 and Table 5. Genetic line and scoring day had an impact on body lesion scores, depending on the rotation, but the interaction between both variables was significant in some cases. Front, middle, and total body lesions decreased over time for both genetic line but not rear body lesions between d26 and d1. Concerning differences between genetic lines, HP2 sows from the rotation 5 had more middle, rear, and total body lesions than HP1, independently of the scoring day. In addition, HP2 sows from rotation 4 had more middle and rear body lesions than HP1 on d26 but they had less front body lesions on d84.

Further analyses indicated some relationships between body lesions and agonistic behaviors. Indeed, the body lesions on the day after mixing were positively correlated with the fight frequency (front: weighted ρ = 0.48, *p* < 0.0001; middle: weighted ρ = 0.33, *p* < 0.0001; rear: weighted ρ = 0.29, *p* < 0.0001; total: and weighted ρ = 0.51, *p* < 0.0001) and the total time spent fighting (front: weighted ρ = 0.51, *p* < 0.0001; middle: weighted ρ = 0.33, *p* < 0.0001; rear: weighted ρ = 0.31, *p* < 0.0001; and total: weighted ρ = 0.49, *p* < 0.0001) on the day of mixing. Despite a weaker association, the frequency of bullying received by sows at mixing was also positively correlated with body lesions on d1 (front: weighted ρ = 0.12, *p* < 0.0001; middle: weighted ρ = 0.21, *p* < 0.0001; rear: weighted ρ = 0.10, *p* = 0.0005; total: and weighted ρ = 0.20, *p* < 0.0001). However, the total non-reciprocal agonistic acts (i.e., bites, knocks, threats, and bullying) received at mixing were not correlated with body lesions at d1 (*p* > 0.10). One month later, the total body lesions on d26 were positively correlated with the subsequent non-reciprocal agonistic acts received on d27 and d29 (data from both days pooled: front: weighted ρ = 0.12, *p* < 0.0001; middle: weighted ρ = 0.16, *p* < 0.0001; and total: weighted ρ = 0.14, *p* < 0.0001).

### 3.3. Individual and Reproductive Performance

**Figure 3:**
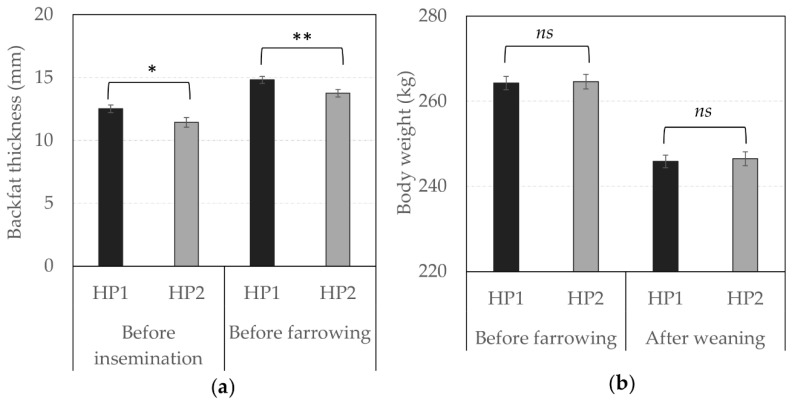
Individual performance according to genetic line. Average (± SEM) (**a**) backfat thickness (in mm) before insemination and before farrowing and, (**b**) body weight (in kg) before farrowing and after weaning in HP1 and HP2. *, ** Means differ significantly (* *p* < 0.050, ** *p* < 0.010) between genetic lines for a same recording phase; *ns*—not significant.

Although body weight did not differ between genetic lines, Figure 3 shows that HP1 sows were characterized by a higher backfat thickness than HP2 sows before insemination (*F*_1,74_ = 4.70, *p* = 0.033) and before farrowing (*F*_1,133_ = 7.11, *p* = 0.009).

**Figure 4:**
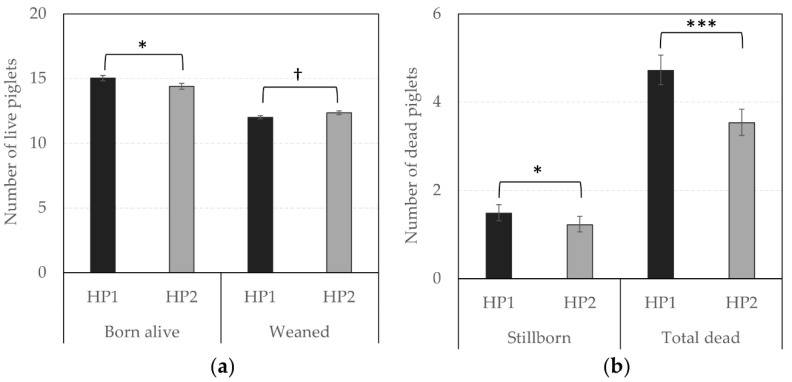
(**a**) Average (± SEM) number of born alive and weaned piglets and, (**b**) Back-transformed predicted mean (CI) number of stillborn and total dead piglets according to genetic line. *, *** Means differ significantly (* *p* < 0.050, *** *p* < 0.001); † Means tend to differ (*p* < 0.10) between genetic lines.

**Figure 5:**
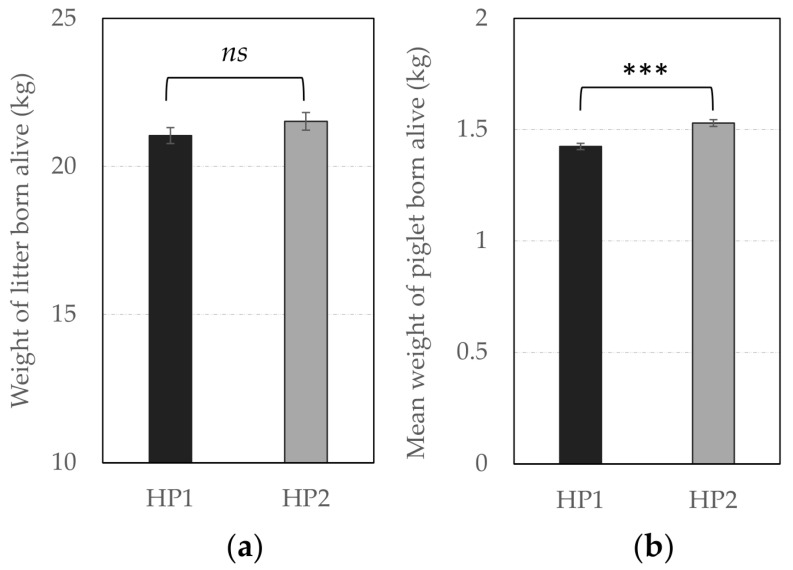
Average weight (± SEM) of (**a**) litter and, (**b**) piglets born alive (in kg) according to genetic line. *** Means differ significantly (*** *p* < 0.001) between genetic lines; *ns*—not significant.

In terms of reproductive performance, the probability of success to farrowing tended to be higher for HP1 than for HP2 sows (OR = 1.31 vs. 1.00, respectively, *F*_1,1238_ = 3.66, *p* = 0.056), indicating that the risk to be removed from the group due to health issues or gestation failure tended to be lower for them. In addition, Figure 4 and Figure 5 illustrate significant differences between offspring of both genetic lines. HP1 sows had bigger litter sizes (i.e., number of alive piglets at birth, *F*_1,225_ = 4.21, *p* = 0.041) but their piglets were lighter than piglets from HP2 sows (*F*_1,224_ = 25.66, *p* < 0.0001), so that the litter weight did not differ between both genetic lines (*p* > 0.10). Nevertheless, HP1 sows had a higher number of stillborn (*F*_1,225_ = 4.07, *p* = 0.045) and total dead (*F*_1,203_ = 28.09, *p* < 0.0001), but they were less likely to give birth to one or several mummified piglets (OR = 0.50 vs. 1.00, respectively, *F*_1,590_ = 13.27, *p* = 0.0003) than HP2 sows. As a result, the number of weaned piglets tended to be lower for HP1 than for HP2 sows (*F*_1,216_ = 3.38, *p* = 0.067).

The individual performance of sows was also modulated by the rotation. Sows from rotation 5 were significantly heavier than sows from rotations 3 and 4 both before farrowing (*F*_2,181_ = 4.09, *p* = 0.018) and after weaning (*F*_2,174_ = 46.35, *p* < 0.0001), and their backfat was also thinner before farrowing (*F*_2,133_ = 12.19, *p* < 0.0001). Reproductive performance varied between rotations (*F*_2,1238_ = 3.21, *p* = 0.041), with sows from rotation 3 less likely to complete the gestation than sows from rotation 5 (OR = 0.62 vs. 1.00, respectively, *F*_1,1238_ = 3.21, *p* = 0.041). However, sows from rotation 3 lost an average of 3.61 (3.35; 3.90) piglets (total number of dead piglets) compared to 4.12 (3.73; 4.56) and 4.56 (4.08; 5.11) piglets for sows from rotations 4 and 5, respectively (*F*_2,203_ = 6.27, *p* = 0.023).

## 4. Discussion

In this study, we investigated the effect of two genetic lines derived from Landrace × Yorkshire sows on the welfare and productivity of sows housed in large semi-static groups in a commercial farm. The results indicate that two independent selection processes within a same breed induced behavioral differences and modulated welfare differently. Aggression and chronic stress are recurrent issues in gestating sow group-housing systems. With the increasing public concern regarding pig welfare and the adoption of new regulations in favor of group-housing, pig breeding companies should now develop low aggression genetic lines adapted for group living.

### 4.1. Impact of Genetic Selection on Agonistic Behavior and Welfare

Aggressiveness of sows is a heritable trait that can be selected inadvertently when developing highly productive genetic lines [9,33]. In both genetic lines, aggression was maximal during the 2 h post-mixing due to hierarchy formation and decreased significantly thereafter [34]. Sows from the HP2 genetic line showed a more aggressive behavioral pattern around mixing (d0 and d2). This outcome was evidenced by higher frequencies of fights and bullying in HP2 sows compared to HP1 sows, independently of the observation day. The total time spent fighting was also higher for HP2 on day 2. The HP1 sows were more frequently observed performing bites and knocks than HP2 at mixing, illustrating a less aggressive strategy compared to HP2 sows that were persistently attacking non responders or retreating pen-mates on this day (i.e., bullying). HP2 sows were more aggressive around mixing while social aspects were considered in the selection of the nucleus sows (i.e., asocial or over-aggressive nucleus sows removed from selection) and not in HP1 genetic line. In addition, nucleus sows from the HP2 breeding company were all housed in groups while part of the nucleus sows from the HP1 breeding company were housed individually. However, it is important to note that social aspects were not part of the formal selection criterion in the breeding scheme in HP2 genetic line. Indeed, this only concerned the exclusion of aberrant social behaviors, meaning that this measure may not have “forced” the selection of calm or non-aggressive animals.

It is generally acknowledged that hierarchy formation lasts between two- and ten-days post-mixing [35,36], but agonistic behaviors still occur occasionally thereafter. While sows were engaged in vigorous fights and bullying at mixing to establish dominance relationships, agonistic interactions were reduced and mostly limited to single bites, knocks, and threats in the established groups one-month post-mixing [37]. Aggression under stable conditions is thought to occur due to competition for resource access (e.g., feeding and resting area), hunger, discomfort, irritability or frustration [5,38]. In this study, HP1 sows delivered more threats, bites, and knocks one month later, at day 29, suggesting that they may experience more stress than HP2 sows. Alternately, this higher rate of non-reciprocal agonistic acts may reflect a certain form of long-term social instability in HP1, since prior studies have shown that low levels of aggression at the time of mixing is a predictor for chronic aggression [39,40]. Nevertheless, the analysis of the body lesions scores did not support the hypothesis of higher aggressivity for HP1 sows one month after mixing since lesions scores were higher in HP2 sows, independently of the gestation stage. The scarcity of agonistic behaviors one-month post-mixing makes it difficult to draw strong conclusions at this stage of gestation. Overall, several aspects of dominance relationships and social structure still need to be elucidated. In their sophisticated study of agonistic interactions in groups of 15 growing pigs, Foister et al. [41] applied social network analysis (SNA), which is a mathematical approach examining sociality from the individual to the group level and quantifying direct and indirect (i.e., “friends of friends”) relationships in social groups [42]. They found that what mattered was the size of the clique (i.e., defined as the number of animals that fought and bullied each other within the group) rather than the level of aggression at the group level. In other words, they suggested that the presence of a large central clique with a clearly established hierarchy would be important for social stability, and that other group members would not necessarily need to be involved directly in aggression. Future research should consider using SNA to explore whether genetic line influences relationships and the extent to which central individuals in the group maintain social cohesion between gestating sows on the long-term. Newly-popularized automatic techniques to detect behaviors [43,44,45,46,47] will help to gather large amounts of data to study the social network of large groups of sows as found in many North American industries.

Agonistic interactions typically result in the accumulation of body lesions that can be used as indicators of aggressiveness [30], and this was highlighted by the positive correlations between agonistic behaviors received by sows and their body lesion scores afterwards. The more the sows were involved in reciprocal fights or the more they were bullied at mixing, the more they had high body lesions scores on day 1. In addition, higher body lesions one month after mixing (day 26) were related to future reception of non-reciprocal acts one or three days after (day 27 and 29, respectively). This latter finding indicates that principal recipients of agonistic interactions within the first month after mixing are also the principal recipients of agonistic interactions later. Analyses of body lesion scores indicated that there was a triple interaction between genetic line, scoring day, and rotation and models were thus run a second time by rotation in order to put the emphasis on the effect of genetic line and scoring day within each rotation. As expected, front, middle, and total body lesions scores were maximal on the day after mixing and they decreased to be minimal on day 84 for both genetic lines. Interestingly, the rear body lesions were higher on day 26 than on the day after mixing in HP2 sows from rotations 3 and 4. While front body lesions were previously found to be the result of reciprocal fights, rear body lesions resulted mostly from non-reciprocal bullying [30,48]. Hence, this suggests that aggression persisted for a longer time in HP2 sows and they were still victims of bullying, including other non-reciprocal acts, between days 2 and 26. The evaluation of lesions also provided important insights on overall aggressiveness between genetic lines, with HP2 sows having more body lesions than HP1. Higher lesion scores of HP2 in rotation 5 concerned the middle and rear body region, as well as the total body lesions (i.e., front, middle, and rear lesions pooled and averaged) recorded at mixing, and one- and three-months post-mixing. Results were less clear-cut in rotation 4 since differences concerned the middle and rear body lesions at day 26 only. Thus, body lesion scores indicate that HP2 sows were more likely to assault pen-mates than HP1 sows. Surprisingly, HP1 sows from rotation 4 had more front body lesions than HP2 sows on day 86. The authors have no knowledge about the reasons that could have caused aggression and resulted in more severe front injuries in these HP1 sows late during gestation. The lack of genetic line effect in rotation 3 means that both genetic lines did not differ at this life stage and differences in body lesions only appeared later. Perhaps the level of aggression decreased faster with age and experience for HP1 sows than HP2 sows, which may be translated by lesion score differences detectable later in life only. It is therefore recommended to study animals over the long-term, during several gestation cycles, to catch the potential delayed effects of genetic selection on animal welfare. On the other hand, unlike lesion scores, behavioral differences were detected independently of rotation number, so including rotations 3 and 4. Caution is thus recommended when evaluating lesion scores as some authors failed to find a relationship between aggressive behavior and lesions [49]. For instance, Karlen et al. [37] argued that agonistic interactions are not the only cause of body damages and sows may also be injured by hurting themselves on pen features or climbing over one another. An alternative explanation would be that the lesion scores scale was not sensitive enough to highlight differences in rotation 3 when the injuries were more severe. This does not preclude that scoring body lesions brought complementary information to video analyses since significant differences were found not only at mixing, but throughout gestation even after the hierarchy was formed [50]. The assessment of body lesion scores between genetic lines is interesting since it offers a rapid evaluation of aggression rate in large groups of pigs and can be performed anytime, even after periods of social stress (e.g., mixing or resource competition), as they are the mark of past aggressions (e.g., [49,51,52]). Body lesions are also suitable candidates for genetic selection against aggression [13,53] and could thus be easily incorporated in selection programs in complement to behavioral observations of aggressive behavior.

### 4.2. Other Effects on Agonistic Behavior and Welfare

The present study had the advantage of being conducted in a commercial farm with performance objectives, but this also meant dealing with other variables that can interfere with treatments effects on behavior and welfare. Rotation number, which is related to the age and experience of the sows (i.e., they get older and more experienced as they move from one rotation to the next), influenced the sows’ welfare. Sows from rotation 3 showed more aggressive behavior than sows in rotations 4 and 5 one month after mixing. This finding corroborates literature that showed that aggressiveness decreased with parity and social experience [54,55].

On another hand, behavioral analyses also indicated that sows from rotation 5 were the most aggressive at mixing, as were sows from rotation 4 at day 2, while it was expected that sows from the third rotation would be significantly more aggressive regardless of gestation phase. However, because there was attrition and no further animal was bought from the groups acquisition, group sizes decreased over time. As a result, sows from the rotation 5 or 4 were housed in smaller groups than sows from the rotation 3, and this may have induced confounding effects. According to Samarakone and Gonyou [56] individual aggression can increase with decreasing group size. Despite a literature with discordant results on the influence of group size on sows’ aggression (for a review, see [34]), it seems that pigs in large groups (i.e., 108 vs. 18 pigs in their study) generally adopt less aggressive social strategies or are more likely to avoid conflicts during mixing [56]. Due to the confounding effects between rotation and group size variables in the present study, the rotation variable was considered as an integrative variable taking into account group size. The authors were comfortable with this, as preliminary analyses, including the group size effect, did not show different effects of genetic line on behavior, welfare, and productivity, compared to final analyses. In addition, group size and rotation effects were balanced between treatments.

### 4.3. Finding the Right Balance between Economic Proficiency and Welfare

Public concern for better welfare is increasing while the pig industry also faces a continuously growing demand for pork meat. This leads breeding companies to create ever more productive genetic lines in the social context of public awareness. Hence, both genetic lines under study were selected based on overlapping sets of criteria for high performance, including reproductive performance measures (e.g., litter size and piglet weight). However, performance differences were still noticed. This offered room to explore whether HP1, which was less aggressive around mixing and had lower lesion scores throughout gestation (i.e., this latter effect concerned rotation 5; thus, higher parities only), could provide a better profitability through a better reproductive performance to the pig industry. In terms of individual performance, HP1 sows had thicker backfat than HP2 sows. Maintaining optimal body conditions in gestating sows is of considerable importance since excess or deficit of backfat leads to reproductive disorders [57,58]. Sows from both genetic lines were below the optimal the level recommended for reaching optimal reproductive performance, i.e., 15 to 22 mm [59], but they had a good reproductive performance since they weaned an average of 12 or more piglets. Dourmad et al. [59] specify that it is still possible to maintain high productivity with lower backfat thickness in gestating sows, provided that extreme situations are avoided. Despite the backfat thickness difference, body weight did not differ between genetic lines, and was more related to the rotation number and thus to their age; i.e., sows were heavier at rotation 5 when they were older than at the rotation 3. Although both genetic lines had a good reproductive output, HP2 sows appeared to have the highest reproductive performance in terms of piglets’ robustness. HP1 sows tended to have a higher probability of success to farrowing than HP2 sows, but the latter were characterized by offspring with higher robustness since their piglets were heavier at birth and more likely to survive until weaning. As a result, the HP2 tended to wean more piglets than HP1 sows, even though their litter sizes were smaller at birth. Hence, although Løvendahl et al. [33] reported that low aggressiveness among sows at mixing was related to improved maternal abilities, it seems that the least aggressive genetic line performed least well in terms of reproduction in this study. Several hypotheses could explain performance differences. The most plausible one is that breeding scheme differences between each supplier may have drove the selection differently. HP1 and HP2 nucleus sows were selected for their high performance in terms of reproduction, growth, robustness, carcass quality and genomics, but the detailed set of criteria used to develop genetic lines differed between the suppliers. Another hypothesis relates to the social environment for selection that differed between the suppliers. Some of the HP1 nucleus sows were housed in individual stalls throughout gestation whilst all HP2 sows were group-housed in pens during gestation. Social aspects were considered in HP2 breeding company, to some extent, but this was not part of the breeding selection program and results described above showed that this did not force the selection of non-aggressive animals. However, social behaviors that confer an advantage over other group members in terms of reproductive performance could have been selected or counterproductive social behaviors could have been counter-selected in HP2 genetic line.

This experimental study highlighted the difficulty in determining the most suitable genetic line, especially regarding ethical concerns. Indeed, while HP2 sows were more aggressive around mixing and sustained higher lesion scores in higher parities than HP1 sows, which negatively affects sow’s welfare, HP1 sows lost more piglets and tended to wean less piglets than HP2 sows, which may decrease economic profitability and also raise ethical issues around piglet suffering. Future studies should explore whether excessive aggressiveness and abnormal behaviors, as well as progeny survival, can be counter-selected without affecting performance. Encouraging studies suggested that the development of calmer genetic lines may not necessarily be detrimental to profitability. For example, Yoder et al. [60] showed that individuals characterized by a less reactive temperament (i.e., less vocal and calmer when separated from pen-mates, loaded and confined in a scale for scanning procedures) were fatter, had greater loin depth, and grew faster. Until now, pig breed companies often largely focused their efforts on genetic selection based on individual production characteristics [11] and traditionally ignored the effects of the (social) environment, which can modulate responses to selection [17]. Regarding the ethical implications of breeding selection and the potential negative effects of pig social stress on pig production and welfare [61], pig breeding companies should now consider integrating the selection of favourable behavioral traits for group living in their breeding schemes.

Selection experiments have successfully developed genetic lines against undesirable behaviors but this type of approach would not be adopted by commercial breeding which are dependent on economic profitability [62], except if welfare traits are directly correlated with production traits [53]. However, selecting animals based on group performance instead of individual performance, and incorporating the indirect genetic effects (IGE) in breeding schemes is a promising avenue for breeding industries [63]. The IGE, also called social genetic effect, competitive or associative effect, is the genetic effect that an individual has on a trait of a social partner [64,65,66,67]. Baud et al. [68] reported that the indirect genetic effect of social behavior explained up to 29% of phenotypic variance for health and disease traits in mice and can thus bias estimates of direct genetic effects. In laying hens, Rodenberg et al. [69] included social effects into a breeding program and were able to develop genetic lines with reduced mortality due to cannibalistic pecking. Experimental research is still in progress in swine but initial studies are promising since genetic selection on high IGE for growth reduced biting behavior and tail damage of growing pigs [66,70].

## 5. Conclusions

The present study aimed at evaluating the aggressiveness and welfare of sows from two genetic lines developed for high reproductive performance in a typical Canadian commercial setting where animals are housed in large semi-static groups. The paper brought important insights on the importance of genetic background on the adaptability of gestating sows to group-housing conditions, but also highlighted the difficulty in determining a most suitable genetic line. Indeed, while one genetic line was more aggressive around mixing and sustained higher lesion scores in higher parities, which negatively affect sow’s welfare, the other produced piglets with lower survivability, which may decrease economic profitability and also raise ethical issues regarding piglets’ welfare. The future challenge of pig breeding companies will be to integrate social effects on productivity into their breeding schemes in order to properly estimate productivity and improve the sows’ adaptability and welfare in group-housing systems.

## Figures and Tables

**Table 1:** Summary of the selection program and housing conditions of nucleus sows in the two independent Canadian suppliers.

Genetic Line	HP1	HP2
Genetic Background	F1 derived from Landrace × Yorkshire	F1 derived from Landrace × Yorkshire
Selection criteria		
Reproduction criteria	yes	yes
Growth criteria	yes	yes
Robustness criteria	yes	yes
Carcass Quality criteria	yes	yes
Genomics	yes	yes
Nucleus sows housing	housed individually or in groups of various sizes	housed in groups of 20–25 animals
Consideration of social aspects	no	only asocial or over-aggressive animals removed from selection, but not part of the formal selection program

**Table 2:** Summary of the impact of genetic line (HP2 vs. HP1), scoring day (d1, d26, and d84) and their interaction on **FRONT** body lesion scores within each rotation (3, 4, and 5).

Fixed Effect	Levels Comparison ^a^	Rotation 3		Rotation 4		Rotation 5	
		OR	*p*-Value ^b^	OR	*p*-Value ^b^	OR	*p*-Value ^b^
Genetic line			(ns)		(0.016)		(ns)
	HP2 vs. HP1	1.00	ns	0.73	0.016	1.22	ns
Scoring day			(<0.0001)		(<0.0001)		(<0.0001)
	d26 vs. d1	0.55	<0.0001	0.71	0.09	0.38	<0.0001
	d84 vs. d1	0.14	<0.0001	0.19	<0.0001	0.06	<0.0001
Genetic line × Scoring day			(ns)		(0.006)		(ns)
	HP2 vs. HP1 at d1			0.62	ns		
	HP2 vs. HP1 at d26			1.10	ns		
	HP2 vs. HP1 at d84			0.58	0.038		
	d26 vs. d1 in HP1			0.53	0.075		
	d84 vs. d1 in HP1			0.20	<0.0001		
	d26 vs. d1 in HP2			0.94	ns		
	d84 vs. d1 in HP2			0.19	<0.0001		

^a^ The reference level of genetic line is HP1 thus odd ratios higher than 1.00 indicate that lesion scores are higher in HP2 than HP1. Similarly, the reference level of scoring day is d1, thus odd ratios higher than 1.00 indicate that scores at d26 or d84 are higher than d1. ^b^ Joint test of fixed effects between parenthesis. ns—not significant.

**Table 3:** Summary of the impact of genetic line (HP2 vs. HP1), scoring day (d1, d26, and d84) and their interaction on **MIDDLE** body lesion scores within each rotation (3, 4, and 5).

Fixed Effect	Levels Comparison ^a^	Rotation 3		Rotation 4		Rotation 5	
		OR	*p*-Value ^b^	OR	*p*-Value ^b^	OR	*p*-Value ^b^
Genetic line			(ns)		(0.070)		(<0.0001)
	HP2 vs. HP1	1.06	ns	1.31	0.070	2.63	<0.0001
Scoring day			(<0.0001)		(<0.0001)		(<0.0001)
	d26 vs. d1	0.61	0.0001	0.96	ns	0.48	<0.0001
	d84 vs. d1	0.32	<0.0001	0.52	0.0004	0.13	<0.0001
Genetic line × Scoring day			(ns)		(0.030)		(ns)
	HP2 vs. HP1 at d1			1.20	ns		
	HP2 vs. HP1 at d26			1.92	0.030		
	HP2 vs. HP1 at d84			0.98	ns		
	d26 vs. d1 in HP1			0.76	ns		
	d84 vs. d1 in HP1			0.57	ns		
	d26 vs. d1 in HP2			1.22	ns		
	d84 vs. d1 in HP2			0.47	0.022		

^a^ The reference level of genetic line is HP1 thus odd ratios higher than 1.00 indicate that lesion scores are higher in HP2 than HP1. Similarly, the reference level of scoring day is dL; thus, odd ratios higher than 1.00 indicate that scores at d26 or d84 are higher than d1. ^b^ Joint test of fixed effects between parenthesis. ns—not significant.

**Table 4:** Summary of the impact of genetic line (HP2 vs. HP1), scoring day (d1, d26, and d84) and their interaction on **REAR** body lesion scores within each rotation (3, 4, and 5).

Fixed Effect	Levels Comparison ^a^	Rotation 3		Rotation 4		Rotation 5	
		OR	*p*-Value ^b^	OR	*p*-Value ^b^	OR	*p*-Value ^b^
Genetic line			(ns)		(ns)		(<0.0001)
	HP2 vs. HP1	1.00	ns	1.24	ns	2.34	<0.0001
Scoring day			(<0.0001)		(<0.0001)		(<0.0001)
	d26 vs. d1	1.30	ns	2.32	<0.0001	1.15	ns
	d84 vs. d1	0.71	0.028	1.27	ns	0.29	<0.0001
Genetic line × Scoring day			(0.052)		(0.091)		(ns)
	HP2 vs. HP1 at d1	0.73	ns	0.96	ns		
	HP2 vs. HP1 at d26	1.30	ns	1.73	0.020		
	HP2 vs. HP1 at d84	1.04	ns	1.14	ns		
	d26 vs. d1 in HP1	0.97	ns	1.73	ns		
	d84 vs. d1 in HP1	0.60	0.066	1.16	ns		
	d26 vs. d1 in HP2	1.73	0.023	3.11	<0.0001		
	d84 vs. d1 in HP2	0.84	ns	1.38	ns		

^a^ The reference level of genetic line is HP1 thus odd ratios higher than 1.00 indicate that lesion scores are higher in HP2 than HP1. Similarly, the reference level of scoring day is d1, thus odd ratios higher than 1.00 indicate that scores at d26 or d84 are higher than d1. ^b^ Joint test of fixed effects between parenthesis. ns—not significant.

**Table 5:** Summary of the impact of genetic line (HP2 vs. HP1), scoring day (d1, d26, and d84) and their interaction on **TOTAL** body lesion scores within each rotation (3, 4, and 5).

Fixed Effect	Levels Comparison ^a^	Rotation 3		Rotation 4		Rotation 5	
		OR	*p*-Value ^b^	OR	*p*-Value ^b^	OR	*p*-Value ^b^
Genetic line			(ns)		(ns)		(<0.0001)
	HP2 vs. HP1	1.03	ns	1.08	ns	2.51	<0.0001
Scoring day			(<0.0001)		(<0.0001)		(<0.0001)
	d26 vs. d1	0.60	0.0001	1.09	ns	0.44	<0.0001
	d84 vs. d1	0.19	<0.0001	0.36	<0.0001	0.08	<0.0001
Genetic line × Scoring day			(ns)		(0.018)		(ns)
	HP2 vs. HP1 at d1			0.84	ns		
	HP2 vs. HP1 at d26			1.63	ns		
	HP2 vs. HP1 at d84			0.91	ns		
	d26 vs. d1 in HP1			0.79	ns		
	d84 vs. d1 in HP1			0.35	0.0005		
	d26 vs. d1 in HP2			1.52	ns		
	d84 vs. d1 in HP2			0.38	0.001		

^a^ The reference level of genetic line is HP1 thus odd ratios higher than 1.00 indicate that lesion scores are higher in HP2 than HP1. Similarly, the reference level of scoring day is d1, thus odd ratios higher than 1.00 indicate that scores at d26 or d84 are higher than d1. ^b^ Joint test of fixed effects between parenthesis. ns—not significant.

## Supplementary Materials

The following are available online at https://www.mdpi.com/2076-2615/10/12/2299/s1, Table S1: Summary of the information related to replicates.
