# Supplementary material for: The Role of Genetic Selection on Agonistic Behavior and Welfare of Gestating Sows Housed in Large Semi-Static Groups
Source: Animals (Basel). 2020 Dec 4;10(12):2299. doi: 10.3390/ani10122299 (PMC7762012; doi:10.3390/ani10122299)
Supplement: Supplementary file 1 [file animals-10-02299-s001.pdf]

animals

MDPI

Table S1. Summary of the information related to replicates.

Replicate	Date	Cohort	Rotation	Group Number	Genetic Line	Parity Range	Group Size
1	2/4/2019	1	3	1	HP1	1–3	89
				2	HP2	1–3	84
2	4/1/2019	3	3	3	HP1	2–3	50
				4	HP2	1–3	64
3	4/29/2019	4	3	5	HP1	2–3	69
				6	HP2	2–3	67
4	5/27/2019	5	3	7	HP1	2–3	80
				8	HP2	2–3	91
5	7/22/2019	2	4	9	HP1	2–4	67
				10	HP2	2–4	54
–6	8/19/2019	3	4	11	HP1	2–4	46
				12	HP2	2–4	61
7	9/16/2019	4	4	13	HP1	2–4	53
				14	HP2	2–4	54
8	11/11/2019	1	5	15	HP1	3–5	61
				16	HP2	3–5	54
9	12/9/2019	2	5	17	HP1	3–5	62
				18	HP2	3–5	46
10	1/6/2020	3	5	19	HP1	3–5	44
				20	HP2	3–5	45

Publisher's Note: MDPI stays neutral with regard to jurisdictional claims in published maps and institutional affiliations.

© 2020 by the authors. Licensee MDPI, Basel, Switzerland. This article is an open access article distributed under the terms and conditions of the Creative Commons Attribution (CC BY) license (http://creativecommons.org/licenses/by/4.0/).
